# Effect of socio-economic factors in utilization of different healthcare services among older adult men and women in Ghana

**DOI:** 10.1186/s12913-016-1661-6

**Published:** 2016-08-16

**Authors:** B. I. I. Saeed, A. E. Yawson, S. Nguah, Peter Agyei-Baffour, Nakua Emmanuel, Edmund Ayesu

**Affiliations:** 1 Mathematics and Statistics Department, Kumasi Polytechnic, Kumasi, Ghana; 2 Department of Community Health, College of Health Sciences, University of Ghana, Korle-Bu, Accra, Ghana; 3 Paediatric Department, Komfo Anokye Teaching Hospital, Kumasi, Ghana; 4 Department of Community Health, College of Health Sciences, Kwame Nkrumah University of Science and Technology, Kumasi, Ghana; 5 Kumasi Polytechnic, Kumasi, Ghana

**Keywords:** Healthcare utilization, Generalized logit, Socio-economic inequities, Ghana

## Abstract

**Background:**

The older adult population is increasing all over the world. In sub-Saharan Africa, due to poverty and low policy coverage, older adults are not well catered for. The effect of socio-economic inequality in the use of healthcare services among older adult men and women in Ghana was investigated in this paper.

**Methods:**

The data employed in the study were drawn from Global Ageing and Adult Health survey SAGE Wave 1 Ghana and was based on the design for the World Health Survey 2003, SAGE Wave 0, Ghana. The survey was conducted in 2007–2008 and collected data on socio-economic characteristics and other variables of the 5573 individuals interviewed.

**Results:**

Using generalized logit model, the study found that health status is a very strong determinant of the type of healthcare services preferred by older adults Ghanaians. Men with higher income preferred the private health facilities, while those who completed tertiary education, those with health insurance and those who self-rated their health as very bad, bad or moderate preferred public facility. Self-employed men and those in informal employment, preferred other health facilities outside the formal public health service. Women with primary and secondary education, preferred the private health facilities. Women with health insurance, those in middle and upper class income quintiles or those with self-rated bad and moderate health status or being relatively younger preferred the public facility to other health services. Self-employed women and those in informal employment preferred traditional treatment. In Ghana, there are important socio-economic gradients in the use of some healthcare services. In both sexes, those without insurance and rural residents preferred the pharmacy and traditional treatment.

**Conclusion:**

These differences may be due to socio-economic inequities but could also indicate that the existing health facilities are not always used in an optimal way. Patient factors may be equally important as supply factors in explaining the differential use of health services. The public health systems in Ghana still have a major role in improving the health of older adults. National commitments in providing basic essential infrastructure and personnel to health centres for the citizenry is imperative. Policy readjustment of the national health insurance scheme to make it truly accessible to the aged is essential.

## Background

The older adult population is increasing all over the world, which may be explained by relatively improved socio-economic and livelihood conditions favourable for longevity such as increased access to healthcare, improving food security, urbanization and general security [1]. However the situation does not reflect a global uniformity in terms of access to health improving facilities, especially in sub Sahara Africa, where due to poverty and low policy coverage, most older adults are not well catered for as compared to their counterparts in the developed world [2]. The situation of most older adults in many developing countries is similar to that of other vulnerable populations. Exclusion with regards to favourable accessibility to basic social infrastructure and food security challenges are a stark characteristic of most older adults in these countries [3, 4]. It is arguably true that socio-economic condition affects health related quality of life and utilization [5, 6]. Recent literature has identified income and production resources as good predictors of quality health [7, 8]. People living on low incomes have been identified as standing higher risk of suffering serious illness and death than those in upper income brackets, such that people with reasonably high savings are less prone to predisposing depressive factors.

In sub-Sahara Africa, the older adults population is exposed to antecedent vulnerabilities including poverty, low coverage of health infrastructure and weak social welfare and support institutions [2, 4]. All these and other socio-cultural factors that define the peculiarity of the situation of older adults in Africa can explain utilization of heath and healthcare products. The measurement of these antecedents and their interactions with health and healthcare utilization are underlying imperatives to designing practical policies for the welfare of the aged population.

In Ghana, traditional medicine practices are a significant part of the Primary Health Care system. The entire body of traditional medicines encapsulates the totality of herbal, spiritual, manual and exercise techniques aimed at treating, diagnosing and preventing diseases or maintaining well-being [9, 10]. It has been estimated that about 80 % of the population of Ghana relies on herbal preparation for Primary Health Care as a result of affordability, easy access and cultural beliefs [10].

The country’s healthcare system can be classified into four main categories. The first category involves public health facilities managed by the Ministry of Health (MOH) including teaching hospitals, regional and district hospitals, clinics, and health posts. The second and third categories include private-for-profit and private not – for – profit biomedicines which are manned by doctors, midwives and pharmacists. The last category is made up of traditional medicine practitioners who practice as established or itinerant entities throughout the country [11]. In terms of personnel capacity, it is estimated that there is a ratio of 1:200 Traditional Medicine Practitioners per capita compared to 1:20,000 medical doctors [9].

In Ghana, the public health facilities and mission hospitals and clinics offer service through the national health insurance scheme NHIS, unlike most private-for-profit facilities where fee for service is the means of payment. Unfortunately most private not – for – profit manned by NGOs, do not offer mainstream health care services-they may offer family planning and other public services [11]. Traditional Medicine Practitioners operate without the NHIS, however systems of payment are flexible and may include non-monetary forms of payments i.e., payment in kind or provision of services to the practitioner [9, 10]. Generally private-for-profit facilities are seen to be more expensive compare to the public and mission facilities. Traditional medicine may the seen as most affordable and accessible.

Studies as well as policies on health and healthcare utilization in Ghana have predominantly focused on gender, rich-poor and rural-urban gaps [12–14]. This might have been informed by the spread and gender inequities in accessibility to healthcare due to infrastructural deficits inherent to the health care system. It has been estimated that the average Ghanaian needs to travel a distance of 16 km to the nearby hospital for healthcare services. Whilst those in the urban centers have a more favourable average distance of five (5) kilometers. Others in poor and deprived rural regions need to traverse further distances on poor road infrastructure to access health services [14]. Financial accessibility is one underlying phenomenon influencing choices of health utilization in the country [15]. Rich households are more likely to utilize modern hospital facilities for healthcare as compared to poor households who are likely to use herbal medicines and self-medication [16].

Introduction of user fees for health care in Ghana (in 1985) resulted in a sharp and significant reduction in utilization of health care, prevented access for the poor, and imposed considerable financial difficulties on the population. It led to delays in seeking health care and reduced access to health care by the extremely poor including the aged [17–19]. Health insurance is seen as an important alternative financing mechanism for health care in developing countries, with the potential to increase utilization and better protect people against (catastrophic) health expenses [20, 21]. The National Health Insurance Scheme was introduced in 2003 and became operational in 2005 to reduce the financial barriers to accessing health care so as to improve the health of the population [20, 22, 23]. It is almost a decade into its implementation and challenges to access to health care still exists [24].

On the whole, research space on healthcare utilization has not given much attention to the older adult population in Ghana. This may be due to the overwhelming majority of the population being youthful [2] and a weak policy focus on older adults. This study is situated within the context of explaining factors that influence utilization of health and healthcare products among older adult men and women in Ghana. It seeks to analyze socio-economic and intrinsic cultural values and how they affect the spans and depth of health utilization in Ghana to influence formulation and implementation of policies on the aged.

## Methods

### Sampling procedures

The data employed in this study were drawn from the World Health Organization Global Ageing and Adult Health, SAGE Wave 1, Ghana [25]. This study seeks to explore the use of different health facilities among older adult Ghanaian men and women in relation to socio-economic factors. Data on SAGE is freely available to the research and policy community. SAGE is a longitudinal study with nationally representative samples of persons aged 50+ years in Ghana with a smaller sample of adults aged 18–49 years. Instruments are compatible with other large high-income country longitudinal ageing studies. Wave 1 was conducted during 2007–2008. In this study, 5573 respondents made up of (2824) men and (2749) women were considered. The main aim is to generate valid, reliable and comparable information on a range of health and well-being outcomes of public health importance, in adult and older adult populations. The face-to-face interview was conducted in Ghana (2008–09). Multistage cluster sampling strategies were used where households were classified into one of two mutually exclusive categories:

1. All persons aged 50 years and older were selected from households classified as ‘50+ households’; and
2. One person aged 18–49 years were selected from a household classified as an ‘18–49 household’.

Household enumerations were carried out in the final sampling units. One household questionnaire was completed per household where a household informant and individual respondent need not be the same individual. One individual was selected from 18 to 49 households, whereas for 50+ households all individuals aged 50+ were invited to complete the individual interview. Proxy respondents were identified for selected individuals who were unable to complete the interview. Household-level analysis weights and person-level analysis weights were calculated for each country, which included sample selection and a post-stratification factor. Post stratification correction techniques used the most recent population estimates provided by the Ghana Statistical Service. A standardized survey instrument, set of methods, interviewer training and translation protocols are used in all SAGE countries.

International standards were used to harmonize education levels and occupations.

### Measures of healthcare services

Information of where most frequently respondent received healthcare over the last 3 years was elicited in one question. The respondents were asked “Think about healthcare you needed in the last 3 years, where did you go most often when you felt sick or needed to consult someone about your health?”

### Socio-economic measures

The measures were age (50–59 (adults) and 60 year and above (Elderly)), and gender. Enabling measures were assessed in terms of education, job employment, insurance, health status and income. Education was recorded as *college/university completed, high school completed, secondary school completed, primary school completed, less than primary school completed and no formal education*. Job employment was categorized into four groups: public, private, self-employed and informal employment. Public sector includes employees of state, or municipal governments and their agencies, parastatal enterprises, and semi-autonomous institutions such as social security institutions that are owned by the government or institutions like religious schools if the staff are paid by the government. Private sector includes any employees not working for the government and not self-employed. Self-employed includes those who earn their livelihood directly from their own trade or business rather than as an employee of another. Informal employment could mean employment in the informal economy or informal employment. Informal economy refers to the general market income category wherein certain types of income and the means of their generation are “unregulated by the institutions of society, in a legal and social environment in which similar activities are regulated.” Jobs in the informal economy are characteristically without benefits such as health insurance, sick leave, paid vacations or pensions. Insurance status was recorded as respondents who have health insurance coverage.

Type of health facility were categorized into Private, Public, Traditional, Charity and Pharmacy. Pharmacy services in this analysis refer to privately owned drug and pharmacy shops and is an independent service from the prescription medicine that patients receive from physicians/ prescribers in a hospital or clinic. Self-rated health status indicates how Ghanaians rated their health state by using a five-point scale ranging from “very good” to “very bad” coded as a categorical variable that ranges from 1 equals “very good” to 5 equals “very bad” . Income level was divided into five categories: Income Quintile; *Q1 (lowest) through Q5 (highest)*. Wealth or income Quintiles were derived from the household ownership of durable goods, dwelling characteristics (type of floors, wells and cooking stove), and access to services (improved water, sanitation and cooking fuel) for a total of 21 assets. A two-step random effects probit model was used to generate the Quintiles.

### Analytical strategy

A generalization of the logistic regression model was applied in the analysis to examine where adult and elderly Ghanaians visited most to receive their healthcare services. Analyses of the association of healthcare services with socioeconomic factors were carried out separately for women and men by means of multinomial logistic regression analysis. Odds ratios (OR) and their respective standard errors were also computed. Firstly, no variable was adjusted for age (age-unadjusted model). Secondly, each variable in the analysis was adjusted for age (age-adjusted model).

For a response with five unordered options (private, charity, traditional, pharmacy and other health services), where public facility was considered reference category due to the highest frequency of reception, the multinomial logistic regression estimates the effects of socioeconomic variables on the different healthcare services with unordered response categories. Stata SE (version 12.1) was used for analysis.

## Results

### Data description

Table 1 presents the descriptive statistics of the data on 5573 respondents used in the current study. Overall, there were slightly more men (50.67 %) than women and more than 50 % were relatively younger (elderly) and (48.84 %) were relatively much older (adults). Table 1 indicated that most of the people preferred to use public health services (44.39 %) while the healthcare service with the least attraction was traditional (2.21 %). A little over half of all respondents were uneducated (50.35 %) and only (2.21 %) respondents completed tertiary education. Almost two-thirds of the respondents had no health insurance coverage (63.59 %). Most respondents were self-employed (81.63 %) and only (8.42 %) were in formal public sector employment. Most respondents self-rated their health as good (38.58 %) and moderate (38.69 %), a few however, self-rated their health as bad (13.10 %) and very bad (2.92 %). Income quintile analysis indicated an almost uniform distribution of a fifth of respondents in each of the five income quintiles.

**Table 1:** Healthcare Utilization by basic demographic and socioeconomic characteristic, SAGE Wave 1, Ghana

Variable	Total	Percentage
Healthcare facility		
Private	569	10.21
Public	2474	44.39
Traditional	123	2.21
Charity	202	3.62
Pharmacy	713	12.79
No use of health services in previous 3 years	1492	26.77
Education		
None	2806	50.35
Primary	1291	23.17
Secondary	1287	23.09
Tertiary	189	3.39
Gender		
Male	2824	50.67
Female	2749	49.33
Insurance		
No	3544	63.59
Yes	2029	36.41
Age category		
Adult	2722	48.84
Elderly	2851	51.16
Employment		
Public	469	8.42
Private	207	3.71
Self-employed	4549	81.63
Informal employment	348	6.24
Health status		
Very good	374	6.71
Good	2150	38.58
Moderate	2156	38.69
Bad	730	13.10
Very Bad	163	2.92
^a^Income		
Quintile1	1081	19.40
Quintile2	1100	19.74
Quintile3	1104	19.81
Quintile4	1127	20.22
Quintile5	1161	20.83

^a^Income Quintile5 indicates the highest income and Quintile1 indicates the lowest income respectively

In addition, Table 2 shows that relatively higher proportion of urban residents used public health facility compared to rural residents (46.60 % vs. 42.89 %) and also used private health facility more (13.68 % vs. 7.81 %). However, compared to urban residents, a higher proportion of rural residents used traditional practitioners (2.46 % vs. 1.84 %) and the pharmacy (14.62 % vs. 10.17 %).

**Table 2:** Healthcare Utilization by location of older adults, SAGE Wave 1, Ghana

Health facility	Urban (%)	Rural (%)
Private	312 (13.68)	257 (7.81)
Public	1063 (46.60)	1411 (42.89)
Traditional	42 (1.84)	81 (2.46)
Charity	77 (3.38)	125 (3.80)
Pharmacy	232 (10.17)	481 (14.62)
No use of health services in previous 3 years	555 (24.33)	935 (28.42)
Total	2281 (100)	3290 (100)

Table 3 demonstrates that, among men, the elderly were less likely to utilize the pharmacy services instead of public services and men in the second income quintile category, were more likely to use private facility compared to those in the least income quintile (Table 4). It is important to note that income and education showed very strong association with utilization of private health facility by men. Those with higher incomes and those who had completed secondary education or higher tended to use the private health facilities.

**Table 3:** Healthcare Utilization by socioeconomic indicators among men (Age –adjusted (except age category) odds ratios (OR) from multinomial logistic regression and their standard errors (SE)), SAGE Wave 1, Ghana

Variables	Private	Charity	Traditional	Pharmacy	Others	(Se)
Age category						
50–60	1	1	1	1	1	
60 and more	1.097 (0.163)	1.058 (0.266)	1.199 (0.359)	0.665^c^ (0.151)	0.800^a^ (0.126)	
Income						
Quintile1	1	1	1	1	1	
Quintile2	2.205^b^ (0.354)	0.731 (0.532)	1.133 (0.606)	1.124 (0.260)	0.946 (0.213)	
Quintile3	2.622^c^ (0.317)	1.200 (0.454)	1.725^b^ (0.412)	0.946 (0.261)	0.888 (0.237)	
Quintile4	2.232^c^ (0.303)	0.912 (0.381)	0.898 (0.395)	0.627^a^ (0.265)	0.735 (0.223)	
Quintile5	4.604^c^ (0.296)	0.426^a^ (0.478)	0.237^b^ (0.614)	0.369^b^ (0.269)	0701 (0.231)	
Education						
Not educated	1	1	1	1	1	
Primary	1.276 (0.224)	0.709 (0.489)	0.899 (0.344)	0.787 (0.189)	0.801 (0.174)	
Secondary	2.075^c^ (0.236)	1.742 (0.368)	0.925 (0.387)	1.105 (0.186)	1.033 (0.160)	
Tertiary	1.909^a^ (0.396)	1.870 (0.694)	0.549 (0.747)	0.371^b^ (0.454)	0.387^c^ (0.333)	
Insurance						
No	1	1	1	1	1	
Yes	0.856 (0.192)	2.602^c^ (0.314)	0.445^c^ (0.302)	0.397^c^ (0.184)	0.568^c^ (0.129)	
Employment						
Public	1	1	1	1	1	
Private	1.837^a^ (0.364)	0.858 (0.629)	0.986 (0.897)	1.621 (0.314)	1.348 (0.338))	
Self-employed	1.502 (0.265)	0.978 (0.417)	2.502^a^ (0.484)	2.521^c^ (0.251)	2.306^c^ (0.188)	
Informal	0.831 (0.540)	2.776^a^ (0.558)	1.608 (0.749)	2.232^b^ (0.376)	2.032^c^ (0.271)	
Health status						
Very good	1		1	1	1	
Good	1.461 (0.435)	0.806 (0.701)	0.437 (0.636)	0.284^c^ (0.268)	0.833 (0.281)	
Moderate	1.318 (0.423)	0.972 (0.699)	0.552 (0.665)	0.242^c^ (0.285)	0.570^b^ (0.271)	
Bad	0.909 (0.488)	2.149 (0.715)	0.913 (0.753)	0.376^c^ (0.307)	0.383^c^ (0.332)	
Very bad	0.869 (0.899)	2.921 (0.956)	0.322 (1.186)	0.203^c^ (0.611)	0.918 (0.483)	

a, b and c indicates 1, 5 and 10 % significance level respectively

**Table 4:** Healthcare Utilization by socioeconomic indicators among women (Age –adjusted (except age category) odds ratios (OR) from multinomial logistic regression and their standard errors (SE)), SAGE Wave 1, Ghana

Variables	Private	Charity	Traditional	Pharmacy	Others
Age category					
50–60	1	1	1	1	1
60 and more	1.123 (0.189)	1.455^a^ (0.222)	1.800 (0.557)	1.021 (0.160)	0.755^b^ (0.118)
Income					
Quintile1	1	1	1	1	1
Quintile2	1.260 (0.280)	1.611 (0.337)	0.448^a^ (0.460)	0.798 (0.217)	0.915 (0.173)
Quintile3	0.940 (0.264)	1.302^a^ (0.331)	0.839 (0.527)	0.389^c^ (0.247)	0.661^b^ (0.172)
Quintile4	1.437 (0.279)	0.798 (0.446)	0.268^b^ (0.548)	0.355^c^ (0.256)	0.578^c^ (0.184)
Quintile5	2.094^c^ (0.248)	1.883 (0.412)	0.339^c^ (1.046)	0.339^c^ (0.287)	0.667^b^ (0.189)
Education					
Not educated	1	1	1	1	1
Primary	1.520^b^ (0.210)	1.045 (0.289)	0.854 (0.396)	0.800 (0.207)	0.699^b^ (0.162)
Secondary	1.351 (0.222)	1.219 (0.339)	0.793 (1.044)	0.412^c^ (0.253)	0.663^b^ (0.178)
Tertiary	1.639 (0.530)	2.231 (0.657)	3.171^c^ (0.313)	0.491 (0.831)	1.174 (0.475)
Insurance					
No	1	1	1	1	1
Yes	0.787 (0.180)	2.213^c^ (0.237)	0.433^b^ (0.411)	0.268^c^ (0.188)	0.653^c^ (0.123)
Employment					
Public	1	1	1	1	1
Private	1.133 (0.635)	0.759 (0.721)	1.039 (0.332)	3.115 (0.699)	0.584 (0.762)
Self-employed	0.918 (0.343)	0.622 (0.355)	199884.8^c^ (0.288)	3.318^b^ (0.575)	3.103^b^ (0.387)
Informal	0.521 (0.500)	1.139 (0.457)	1391009.1^c^ (0.560)	2.256 (0.635)	1.199 (0.477)
Health status					
Very good	1	1	1	1	1
Good	2.316 (0.611)	1.001 (1.050)	0.064 (0.861)	0.764 (0.461)	0.614 (0.405)
Moderate	1.845 (0.615)	1.554 (1.045)	0.082^c^ (0.804)	0.532 (0.451)	0.381^b^ (0.387)
Bad	2.228 (0.665)	1.853 (1.060)	0.227^c^ (0.823)	0.756 (0.505)	0.429^b^ (0.423)
Very bad	2.134 (0.785)	0.369^b^ (0.417)	0.187^a^ (1.068)	0.700 (0.554)	0.833 (0.493)

a, b and c indicates 1, 5 and 10 % significance level respectively

The most elderly among the men tended to patronize charity facility compared to the public health facility (although they had insurance). However, among all men, those with health insurance were less likely to utilize traditional treatment. Those who completed tertiary education were less likely to use the pharmacy compared to public facility. In addition, men in informal employment and the self-employed preferred the use of pharmacy. Men who self-rated their health to be very bad, bad, or moderate preferred public facility.

In summary, men with higher income preferred the private health facilities, while those who completed tertiary education, those with health insurance and those who had worse self-rated health preferred public facility. Self-employed men and those in informal employment, preferred other health facilities outside the formal public health service.

Similar to men, women in the highest income bracket (Q5) preferred private to public facilities. Women who completed primary education or higher preferred the use of private facilities. This was in contradistinction to the findings among men, the difference in education and preference for a particular choice of health facility among men and women was not significant. Self-employed women and those in the informal employment sector preferred traditional treatment. However, women with health insurance, those in higher income brackets (Q4 and Q5) or those with worse self-rated health status preferred public facility. In addition, women who were self-employed preferred the use of the pharmacy.

Among women, traditional treatments was statistically significantly associated with all studied variables except age; it should be noted however, that the proportion of respondents who used traditional medicine was relatively small in this analysis.

In summary, women with primary and secondary education, preferred the private health facilities. Those with health insurance, those in middle and upper class income quintiles or those with worse self-rated health status or the relatively younger older women preferred the public facility. Self-employed women and those in informal employment preferred traditional modes of treatment.

## Discussion

Among men, the elderly prefers attending public health facilities to attending pharmacy stores for treatment as compared to their adult counterparts. In contrast to men, elderly women preferred public facilities to other health services for treatment as compared to their adult counterparts. Other studies have confirmed the preference of orthodox (public facility) to alternative health practices [26]. This may also be supported by another study that relatively improved socio-economic and livelihood conditions are favorable for longevity such as increased access to healthcare [1].

Men in the upper income class preferred public health services. Interestingly, women in the middle to upper levels of income also preferred public health services to pharmacy usage. In addition women in the middle to upper classes preferred public health services to other health services which was unlike the case in men. This is in clear support of previous literature that socio-economic condition, income and resources affect health related quality of life and utilization [5–8]. Low income, inadequate or unavailable pensions have been identified as a major risk for illness and death in older persons [5, 6]. Financial accessibility is one underlying phenomenon influencing choices of health utilization in the country [15]. Older men and women with insurance were noticed to prefer charity organizations for health care. This may potentially indicate the preference for a particular public and social service provided by these organizations who are mainly non-governmental (i.e., not-for-profit organizations) or that the challenges in the public sector (e.g., long waiting times) may be accounting for this interesting finding.

The National Health Insurance Scheme (NHIS) of Ghana was introduced to overcome the financial burden on families and households. However there are critical challenges inhibiting universal coverage as well as limitation on the care for persons 60 years and above who were not in formal sector employment [20, 24, 27]. To improve access to health care among older persons in Ghana, this policy should be looked at.

Analysis of education indicated that among the sexes those with some level of education (and by implication being previously or currently employed) preferred the private health facilities; probably because they could afford the cost of health care. The public sector in Ghana is beset with poor infrastructure, misdistribution of facilities and limited skilled personnel [11]. Therefore older persons who can afford to pay for service go to the private sector where they may been seen early and would not have to join long queues. Access to public and private health facilities are especially worse in the rural parts of the country [14]. It is therefore not surprising to see that relatively higher proportion of urban residents used the public facilities compared to rural residents-primarily as a results of barriers to access. National efforts at improving geographical access to public sector health services especially in deprived areas of the country is a national health policy that requires urgent attention.

The analysis clearly indicates that men and women with health insurance preferred the public health services and those who are self-employed or in informal employment preferred traditional treatment and the pharmacy. Although it is estimated that about 80 % of the population of Ghana relies on herbal preparation for Primary Health Care as a result of affordability, easy access and cultural beliefs. This analysis however, found relatively lower proportions for the use of traditional treatment, probably due to under-reporting by respondents. SAGE Wave 1, relied on self-report and had no means of verifying the type of health care system respondents used. Interestingly, a relatively higher proportion of rural older persons preferred traditional treatment and the pharmacy. The traditional sector is beset with poor regulation and licensing of practitioners which affect the health of patrons [11]. The health of the rural older adult Ghanaian cannot be left entirely in this poorly regulated health sector. This observation strengthens the need for a policy revision to include all older persons above 60 years in the NHIS in Ghana, irrespective of whether the older person was in public sector employment or not.

A previous study established that health utilization was independent of employment status such that regardless of the working condition of the aged, his or her willingness to use alternative health practices instead of orthodox physician was fixed [26]. This was not in conformity with our analysis, where self-employed male and female preferred the pharmacy and other health services to the formal public services. Being employed currently or previously may afford the older adult in Ghana ability to procure care at the most convenient facility, especially in the rural areas the pharmacy or drug stores may be the only available and most convenient source of health care.

It is interesting to note that despite the challenges in the public health sector, older adult men and women who self-rated their health care as very bad, bad or moderately good preferred it as opposed to the other avenues for health care. This may indicate the confidence the population has in the public sector, that critical health care need may be available in the public health centres. This may be especially so in the district capitals, regional and national levels where curative and specialist care are mostly available in public or Government owned health centres. Measures to improve the public sector health system is imperative and improving geographical access to health care in underserved areas of the country will garner improved health and social wellbeing of the older adult Ghanaian.

### Limitation

A major limitation of the study is that, its cross-sectional nature, did not permit cause and effect examination. Marital status, physical exercise and certain cultural factors which could play a role in the choice and utilization of health services as indicated in the literature elsewhere, were not included in this analysis.

## Conclusion

The analysis provides substantial grounds for further research on the interplay between the effect of socioeconomic factors and receiving healthcare services among the aged. It is clear from the findings that the public health systems in Ghana is still a preferred choice and play major role in improving the health of older adults in both rural and urban locations. National commitments in providing basic essential infrastructure and personnel to health centres for the citizenry is imperative. Policy readjustment of the national health insurance scheme to make it truly accessible to the aged is essential.

## Abbreviations

MOH: Ministry of health
NGO: Non-governmental organization
NHIS: National health insurance scheme NHIS
OR: Odds ratio
QI: Income quintile
SAGE Wave 0: World health survey
SAGE Wave 1: Global ageing and adult health survey
WHO: World Health Organization
